# SARS-CoV-2 Vaccine Efficacy in Patients with Hematologic Malignancies: Practical Points for Further Research

**DOI:** 10.1093/oncolo/oyac173

**Published:** 2022-08-25

**Authors:** Deniz C Guven, Taha K Sahin, Serkan Akın, Fatih M Uckun

**Affiliations:** Department of Medical Oncology, Hacettepe University Cancer Institute, Ankara, Turkey; Department of Internal Medicine, Hacettepe University Faculty of Medicine, Ankara, Turkey; Department of Medical Oncology, Hacettepe University Cancer Institute, Ankara, Turkey; Bone Marrow Transplantation Unit, Hacettepe University Cancer Institute, Ankara, Turkey; Immuno-Oncology Program and COVID-19 Task Force, Ares Pharmaceuticals, St. Paul, MN, USA

## Abstract

This letter to the editor responds to comments on a recently published article on seroconversion rates after COVID-19 vaccination.

We thank Sriwijitalai and Wiwanitkit^1^ for commenting on our article and giving us an opportunity to discuss our findings further.^2^ The authors stated that although the immune responses to the SARS-CoV-2 vaccines could be impaired in patients with cancer, the possibility of asymptomatic COVID-19 before or after vaccination could interfere with the results. It should be noted that we specifically focused on seroconversion rates after vaccination in our paper.^2^ However, the elements of the adaptive immune system, including B-cells, CD4+ T cells (especially T helper cells), and CD8+ T cells, play pivotal roles for the course, severity, and health outcomes in patients with COVID-19.^3^ SARS-CoV-2-specific T-cell responses and rapid development of SARS-CoV-2 cross-reactive CD4+ T cells are critically important for the resolution of COVID-19 without major complications following primary infection by directing both the cellular and humoral immunity.^4^ Therefore, further research should focus on measuring T-cell activity in addition to antibody responses to better define protection level against COVID-19 infection.

While we agree that the seronegative patients with cancer still have some level of protection and immunity against COVID-19,^5^ we think that the development of an antibody response to vaccination is still very important in patients with cancer for several reasons. First of all, it was previously demonstrated that the neutralizing antibody response was highly predictive of immune protection against symptomatic COVID-19.^6^ Additionally, Dispinseri et al. observed better survival with the development of an antibody response against SARS-CoV-2 earlier during the infection,^7^ further supporting the surrogacy of antibody responses in COVID-19 protection. COVID-19 could create a mildly symptomatic and asymptomatic clinic in vaccinated patients. While the vaccination significantly reduced COVID-19 risk, two-thirds of the breakthrough infections were still symptomatic in a wide-scale study conducted on healthcare workers.^8^ In contrast to the general population, the breakthrough COVID-19 infections could still lead to significant mortality and morbidity in patients with cancer,^9,10^ pointing out a need for additional measures for COVID-19 prevention in patients with cancer. Finally, other than increased COVID-19 mortality and morbidity, COVID-19 infection could lead to disruptions and delays in cancer care.^11,12^ This issue could be particularly important for patients with hematological malignancies due to persistent viral shedding in these patients.^13^ Therefore, even the protection of asymptomatic or mildly symptomatic COVID-19 infection is vital for patients with cancer, especially in patients under active treatment.

In conclusion, although the vaccination against COVID-19 is vital in patients with hematological malignancies, further research is needed for patients who did not develop seroconversion to prevent COVID-19 morbidity and mortality and COVID-19 related disruptions in cancer care.

## References

[1] Sriwijitalai W , Wiwanitkit V. Therapy in patients with hematologic malignancies, seroconversion rates, and SARS-CoV-2 vaccination. Oncologist. 2022;27:e916. 10.1093/oncolo/oyac169 PMC9632302 36018054

[2] Guven DC , Sahin TK, Akın S, Uckun FM. Impact of therapy in patients with hematologic malignancies on seroconversion rates after SARS-CoV-2 vaccination. Oncologist. 2022;27(4):e357-e361. 10.1093/oncolo/oyac032. 35274729 PMC8982368

[3] Sette A , Crotty S. Adaptive immunity to SARS-CoV-2 and COVID-19. Cell. 2021;184(4):861-880. 10.1016/j.cell.2021.01.007. 33497610 PMC7803150

[4] Le Bert N , Tan AT, Kunasegaran K, et al. SARS-CoV-2-specific T cell immunity in cases of COVID-19 and SARS, and uninfected controls. Nature. 2020;584(7821):457-462. 10.1038/s41586-020-2550-z. 32668444

[5] Ehmsen S , Asmussen A, Jeppesen SS, et al. Antibody and T cell immune responses following mRNA COVID-19 vaccination in patients with cancer. Cancer Cell. 2021;39(8):1034-1036. 10.1016/j.ccell.2021.07.016. 34348121 PMC8313483

[6] Khoury DS , Cromer D, Reynaldi A, et al. Neutralizing antibody levels are highly predictive of immune protection from symptomatic SARS-CoV-2 infection. Nat Med. 2021;27(7):1205-1211. 10.1038/s41591-021-01377-8. 34002089

[7] Dispinseri S , Secchi M, Pirillo MF, et al. Neutralizing antibody responses to SARS-CoV-2 in symptomatic COVID-19 is persistent and critical for survival. Nat Commun. 2021;12(1):2670. 10.1038/s41467-021-22958-8. 33976165 PMC8113594

[8] Bergwerk M , Gonen T, Lustig Y, et al. Covid-19 breakthrough infections in vaccinated health care workers. N Engl J Med. 2021;385(16):1474-1484. 10.1056/NEJMoa2109072. 34320281 PMC8362591

[9] Heudel P , Favier B, Solodky ML, et al. Survival and risk of COVID-19 after SARS-COV-2 vaccination in a series of 2391 cancer patients. Eur J Cancer. 2022;165:174-183. 10.1016/j.ejca.2022.01.035. 35245864 PMC8828434

[10] Schmidt AL , Labaki C, Hsu CY, et al. COVID-19 vaccination and breakthrough infections in patients with cancer. Ann Oncol. 2022;33(3):340-346. 10.1016/j.annonc.2021.12.006. 34958894 PMC8704021

[11] Pinato DJ , Tabernero J, Bower M, et al. Prevalence and impact of COVID-19 sequelae on treatment and survival of patients with cancer who recovered from SARS-CoV-2 infection: evidence from the OnCovid retrospective, multicentre registry study. Lancet Oncol. 2021;22(12):1669-1680. 10.1016/S1470-2045(21)00573-8. 34741822 PMC8565932

[12] Guven DC , Sahin TK, Yildirim HC, et al. Newly diagnosed cancer and the COVID-19 pandemic: tumour stage migration and higher early mortality. BMJ Support Palliat Care. 2021;bmjspcare-2021-003301. 10.1136/bmjspcare-2021-003301 34711656

[13] Goubet A-G , Dubuisson A, Geraud A, et al. Prolonged SARS-CoV-2 RNA virus shedding and lymphopenia are hallmarks of COVID-19 in cancer patients with poor prognosis. Cell Death Differ. 2021;28(12):3297-3315. 34230615 10.1038/s41418-021-00817-9 PMC8259103

